# Reconfigurable magnon interference by on-chip dynamic wavelength conversion

**DOI:** 10.1038/s41598-023-31607-7

**Published:** 2023-03-24

**Authors:** Md Shamim Sarker, Lihao Yao, Hiroyasu Yamahara, Kaijie Ma, Zhiqiang Liao, Kenyu Terao, Siyi Tang, Sankar Ganesh Ramaraj, Munetoshi Seki, Hitoshi Tabata

**Affiliations:** 1 Department of Electrical Engineering and Information Systems, Graduate School of Engineering, The University of Tokyo, 7-3-1 Hongo, Bunkyo-ku, Tokyo, 113-8656 Japan; 2 Department of Bioengineering, Graduate School of Engineering, The University of Tokyo, 7-3-1 Hongo, Bunkyo-ku, Tokyo, 113-8656 Japan; 3 Institute of AI and Beyond, The University of Tokyo, 7-3-1 Hongo, Bunkyo-ku, Tokyo, 113-8656 Japan; 4 Center for Spintronics Research Network, Graduate School of Engineering, The University of Tokyo, 7-3-1 Hongo, Bunkyo-ku, Tokyo, 113-8656 Japan; 5 Department of Electrical and Electronic Engineering, Khulna University of Engineering and Technology, Khulna, 9203 Bangladesh

**Keywords:** Electronic and spintronic devices, Spintronics

## Abstract

Spin waves (SWs), an ultra-low power magnetic excitation in ferro or antiferromagnetic media, have tremendous potential as transport less data carriers for post-CMOS technology using their wave interference properties. The concept of magnon interference originates from optical interference, resulting in a historical taboo of maintaining an identical wavevector for magnon interference-based devices. This makes the attainment of on-chip design reconfigurability challenging owing to the difficulty in phase tuning via external fields. Breaking the taboo, this study explores a novel technique to systematically control magnon interference using asymmetric wavevectors from two different SW modes (magnetostatic surface SWs and backward volume magnetostatic SWs) in a microstructured yttrium iron garnet crossbar. Using this system, we demonstrate phase reconfigurability in the interference pattern by modulating the thermal landscape, modifying the dispersion of the interfering SW modes. Thus, we manifest that such a tunable interference can be used to implement reconfigurable logic gates operating between the XNOR and XOR modes by using symmetric and asymmetric interference, respectively.

## Introduction

Spin waves (SWs) are the collective excitation of electron spin propagating through low-damping magnetic materials (e.g., yttrium iron garnet (YIG), CoFeB, NiFe, and Heusler compounds) as waves^1^. Their collective nature enables them to coherently travel a significantly long distance at room temperature. It offers wave-based ultralow-power data transmission capabilities and a wide range of frequency operations spanning from a few GHz (in ferromagnets)^2^ to several tens of terahertz (in anti-ferromagnets)^3^. SWs offer data encoding capability in both amplitude and phase^4–8^. The coherent and long-distance propagation properties of SWs render them suitable for interference-based compact logic devices^9^. Most magnonic devices originate from the optical domain, where light with different wavevectors cannot interfere with each other. Therefore, the preconception of wave interference caused researchers to work with identical wavevectors in the case of magnon interference. This made device engineers focus only on the phase information to additionally reconfigure the devices^10^*.* This limits the capability of device engineers to proceed toward on-chip reconfigurable magnonic devices owing to additional microwave signal sources with identical wavevectors and variable phases, which cannot be easily achieved owing to the structural complexity and difficulty in phase tuning by external fields. However, interference in magnonic devices occurs in terms of perturbation of one magnetic flux (owing to propagating SWs) by another inside the magnetic medium, which fundamentally differs from optical interference (that does not require a medium). Therefore, SWs can interfere with each other irrespective of their wavelength within a specific range and provide constructive and destructive interference features. This may provide the flexibility to control interference by controlling the wavelength, which can easily be modified by external fields (e.g., voltage^11,12^, current^13,14^, strain^15,16^, light^17–19^ etc.) according to the dispersion relationships. This study entailed the development of an SW interference device with different wavevector relationships. To address the different wavevectors, we used two modes of SWs with different dispersion relationships [e.g., magnetostatic surface SWs (MSSWs) and backward volume magnetostatic SWs (BVMSWs)] in a magnetic crossbar structure. Such structures were previously investigated considering identical wavevectors^20–22^*,* which implies rigidity in the on-chip design. Regarding reconfigurability, we reconfigured the thermal landscape of the device to tune the wavevectors of the interfering MSSWs and BVMSWs. The wavevector distribution of BVMSWs and MSSWs follow opposite trends with temperature change in the dipolar-coupled SWs regime, which can be used to implement systematic reconfigurability in our proposed hybrid magnon interference device. In symmetric interference, where the wavevectors of the MSSWs and BVMSWs are identical, destructive interference occurs at 180° phase difference and can function as an XNOR gate. However, by operating in the asymmetric wavevector region (with a certain asymmetry), the position of destructive interference can be moved to a 360° phase difference, where it functions as an XOR logic gate.

**Figure 1:**
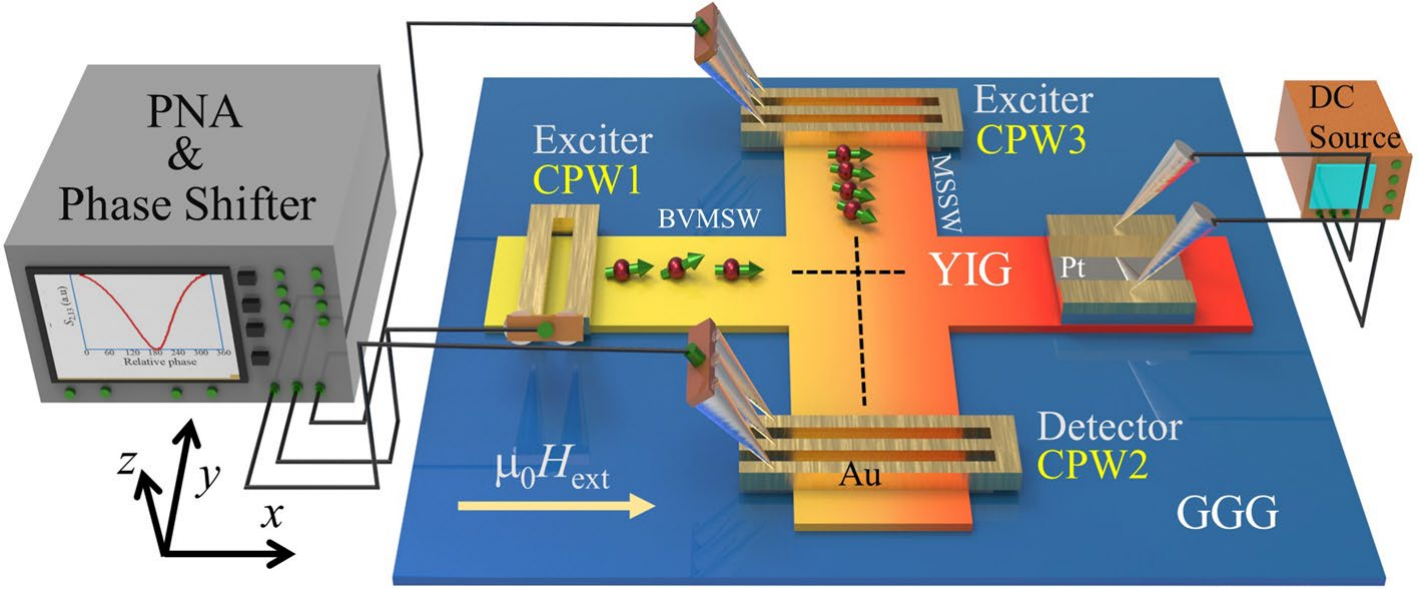
Schematic representation of fabricated device and the measurement systems. The PNA monitor shows typical interference behavior.

A schematic of the measurement system with the crossbar interference device is shown in Fig. 1. The arm of the crossbar placed along the *x*-axis (connected to coplanar waveguide 1 (CPW1)) is referred to as the horizontal bar that supports the BVMSWs. The other arm was placed in a plane but in perpendicular direction to the horizontal bar, referred as the vertical bar, which supports the MSSWs. The length and width of the horizontal bar were 300 and 65 μm, respectively, whereas the length and width of the vertical bar were 450 and 40 μm, respectively. SWs propagate along the orthogonal arms and cause interference. Depending on the wavelengths of the BVMSWs and MSSWs, constructive and destructive interference occur in certain relative phases. For an identical wavevector, where the wavelengths of the interfering SWs are the same, destructive interference occurs at 180°. However, the phase position of the destructive interference shifts as the wavelength of the interfering SWs changes. To introduce reconfigurability, we modulated the wavelength of the propagating SWs by controlling the magnetization of YIG by using a small DC heater with a Pt layer on the YIG surface. The small DC current through the Pt layers results in increased temperature (in the entire crossbar), and a temperature gradient along the SWs propagation path. The increased temperature and the temperature gradient convert the wavelength of the propagating SWs, which is equivalent to modulation of the SW phase. Thus, the position of destructive interference is modified according to the modulated wavelength of the propagating SWs.

## Results and discussion

The basic structure of the proposed reconfigurable SWs interference device along with the measuring system is shown in Fig. 1. The magnetic crossbar comprises of a 90 nm-thick single-crystalline microstructure YIG (Y_3_Fe_5_O_12_) thin film. The X-ray diffraction (XRD) peak and an optical image of the investigated device is shown in the Supplementary material S1. Two antennas (CPW1 and CPW3) were used for SWs excitation, whereas another antenna (CPW2) was used for SWs detection in terms of inductive voltage measurement. Electrical measurements were performed using microwave characterization techniques with a 4-port vector network analyzer (VNA; Model: PNA Network Analyzer N5222B 10 MHz–26.5 GHz). To investigate SWs propagation, we followed an approach similar to that in the literature^23–25^, where transmitted SW spectra were obtained by recording the scattering parameters (*S*_11_, *S*_33_, *S*_21_, *and S*_23_). The direction of the bias magnetic field is maintained along the horizontal bar. MSSWs and BVMSWs, originating from CPW3 and CPW1, respectively, interfere at the cross-junction and induce a change in the magnetic flux, which further reaches CPW2 and is subsequently detected. The output signal intensity reaches a maximum when the propagating waves interfere in-phase (constructive interference). However, the signal intensity becomes minimum when they interfere out-of-phase with each other (destructive interference). The phase of BVMSW is fixed, while the phase of MSSW is tuned from 0° to 360° to enable constructive (at 0° and 360° phase difference) and destructive (at 180° phase difference) interference. The PNA monitor screen in Fig. 1 shows the typical behavior of the interference signal intensity at CPW2 owing to the interference of the incoming SWs originating from CPW1 and CPW3 (*S*_2,13_). This can be obtained by setting following equation in the measurement system:

$$\mathrm{Interference \; gain }=10\times \mathrm{log} \left(\frac{{P}_{{2}_{out}}}{{P}_{{1}_{in}}+{P}_{{3}_{in}}}\right)$$

where ${P}_{{2}_{out}}$ is the detected SWs power at CPW2 (port 2), while ${P}_{{1}_{in}}$ and ${P}_{{3}_{in}}$ are the SWs excitation power at CPW1 (port 1) and CPW3 (port 3), respectively.

**Figure 2:**
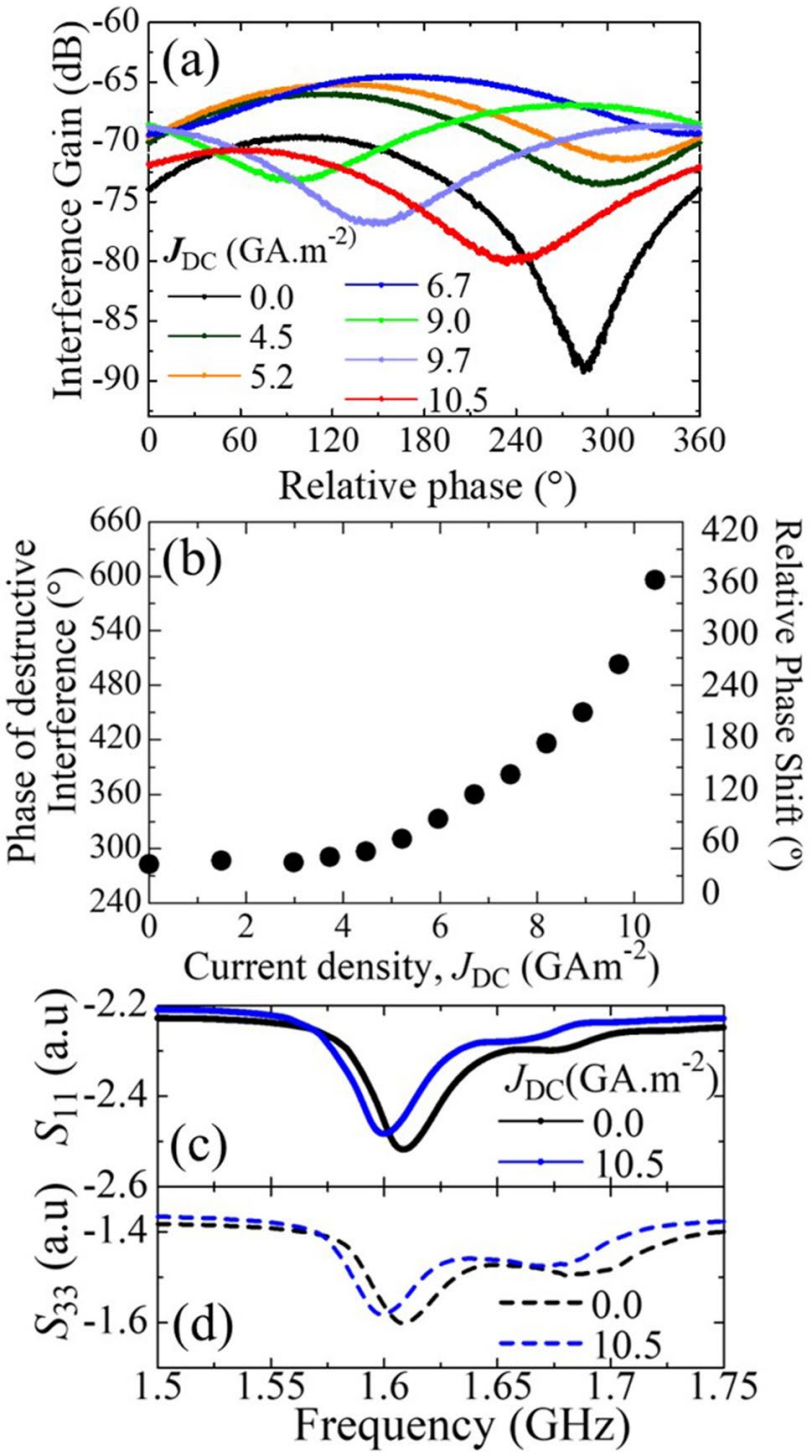
Characterization of interference gain. (**a**) Current density-dependent interference patterns of crossbar device. (**b**) *J*_DC_-dependent shift of the destructive interference position. (**c**) *S*_11_ and (**d**) *S*_33_ parameter of the device at *J*_DC_ = 0 GA m^−2^ (black line) and *J*_DC_ = 10.5 GA m^−2^ (blue line).

We first characterized the interference gain of the proposed crossbar device depending on the applied current density (*J*_DC_) in the Pt heater, as shown in Fig. 2a. We applied a bias magnetic field of μ_0_*H*_ext_ = 18 mT along the horizontal bar to excite MSSWs and BVMSWs in the vertical and horizontal bars, respectively, at a frequency of 1.685 GHz. The SWs wavelengths in each arm determine the interference pattern in the absence of an applied current (i.e., the relative phases for constructive and destructive interference). The interference pattern of the magnetic crossbar with the application of *J*_DC_ = 0, 4.5, 6.0, 7.5, 9.0, 9.7, and 10.5 GA m^−2^, is shown in Fig. 2a (see Supplementary material S2 for a more detailed figure). Destructive interference in the absence of wavelength conversion (*J*_DC_ = 0 GA m^−2^) occurred at approximately 280°. However, as the current density gradually increased, the location of the destructive interference shifted to a higher phase difference of the input waves. It reaches almost 360° at *J*_DC_ = 6.7 GA m^−2^ (see Supplementary Material S1). Thereafter, the phase difference wrapped around, restarting at 0° and again trending upwards to repeat the cycle. The Fig. 2b indicates that the phase change at a lower current density is slower and increases significantly with a higher current density.

We then analyzed the scattering parameters to investigate the mechanism of the destructive interference position shift. We obtained the ferromagnetic resonance (FMR) spectra at ${\mu }_{0}{H}_{\mathrm{ext}}=18\;\mathrm{ mT}$ in terms of *S*_11_ and *S*_33_, as shown in Fig. 2c,d. Figure 2c,d represent the reflection parameters *S*_11_ and *S*_33_ with *J*_DC_ = 0 GA m^−2^ (black curve) and *J*_DC_ = 10.5 GA m^−2^ (blue curve). In the *S*_11_ spectra, *J*_DC_ = 10.5 GA m^−2^ reduces the FMR frequency by 9.4 MHz compared to *J*_DC_ = 0 GA m^−2^. This reduction results from a decrease in the saturation magnetization (*M*_s_) of the YIG at the location of CPW1 because of the change in the current-induced thermal landscape. The relationship between temperature (*T*) and *M*_s_ can be approximated as linear in a temperature range of 300–400 K. This linear dependency up to 400 K has been demonstrated in previous research^26^, and can be modeled as^17^:

1 $${M}_{\mathrm{s}}\left(T\right)\approx {M}_{\mathrm{s},\mathrm{RT}}-\eta \left(T-{T}_{\mathrm{RT}}\right),$$

where ${M}_{\mathrm{s},\mathrm{RT}}$ is the room-temperature saturation magnetization (${M}_{\mathrm{s},\mathrm{RT}}=$ 140 kA m^−1^) and η is the fitting parameter (η = 313 AK^−1^ m^−1^). We measured the temperature-dependent saturation magnetization of the YIG film using superconducting quantum interference measurement, as shown in Supplementary Material S3. The *J*_DC_-dependent quantitative frequency shift due to the current-induced magnetization change was calculated by incorporating the current-induced temperature change (proposed by Thiery et. al.^27^) onto a modified Kittel equation and found to be in good agreement with experimental observations as shown in Supplementary Material S4. An increase in temperature decreased the saturation magnetization and shifted the dispersion relation of the BVMSWs and MSSWs, resulting in wavelength change at the same excitation frequency.

**Figure 3:**
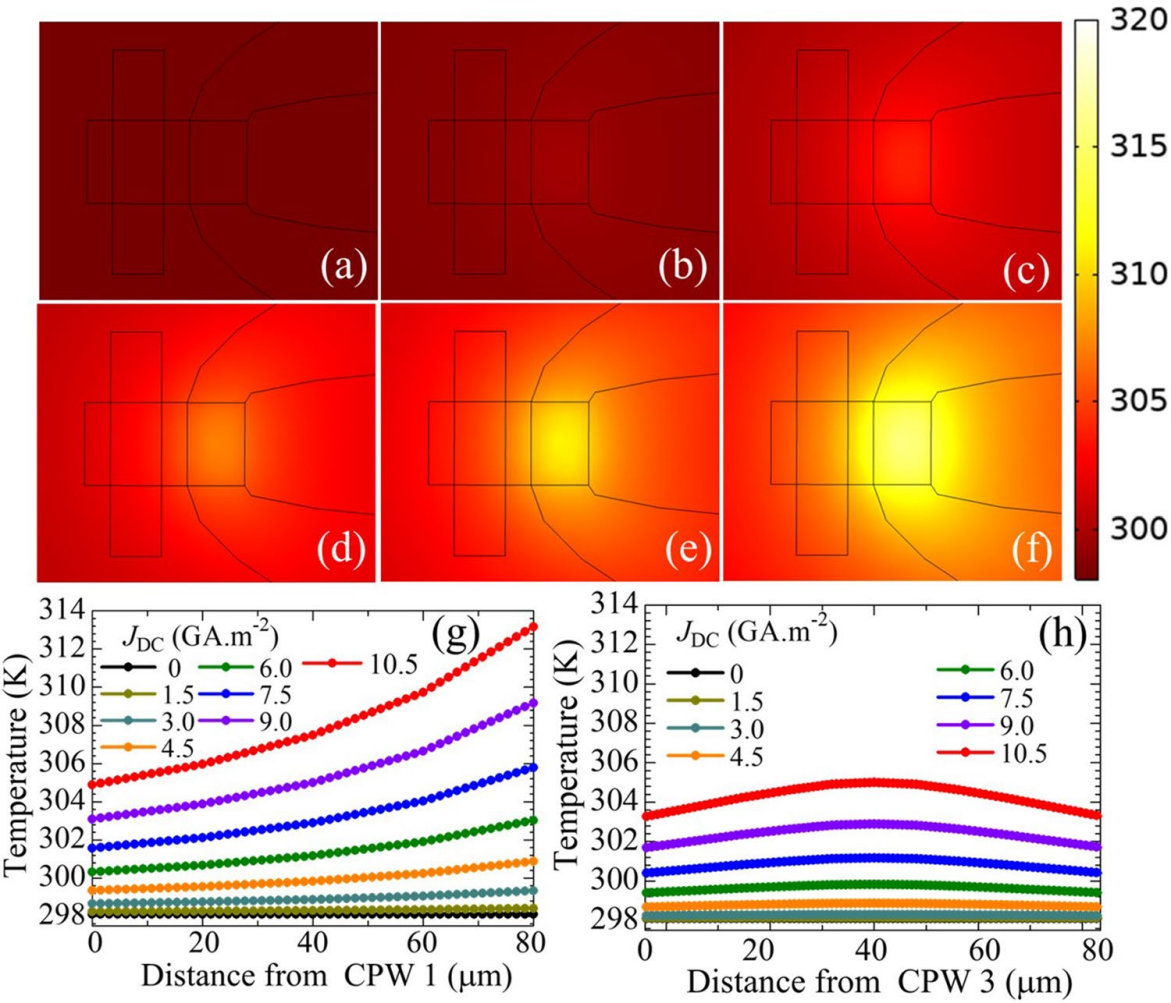
Temperature distribution at (**a**) *J*_DC_ = 0, (**b**)* J*_DC_ = 3.0, (**c**)* J*_DC_ = 6.0, (**d**)* J*_DC_ = 7.5, (**e**)* J*_DC_ = 9.0, and (**f**)* J*_DC_ = 10.5 GA m^−2^. Resulting* J*_DC_-dependent temperature gradient along (**g**) horizontal arm and (**h**) vertical arm of the YIG crossbar.

We calculated the thermal distribution of the YIG crossbar owing to local heating using COMSOL Multiphysics. Figure 3a–f show the temperature distributions for *J*_DC_ = 0, 3.0, 6.0, 7.5, 9.0, and 10.5 GA m^−2^, respectively. The detailed calculation method is included in Supplementary Material S5. A clear shift and a temperature gradient from the heater to CPW1 along the horizontal bar were observed. The temperature along a line through the center of the surface of the horizontal bar is plotted for different *J*_DC_ in Fig. 3g. The *x*-axis represents the horizontal distance from CPW1 to the Pt heater edge. The temperature at CPW1 increased from room temperature (298.1 K) to 304.7 K corresponding to a 6.6 K increase when the applied current density was increased from *J*_DC_ = 0 to 10.5 GA m^−2^. This matches the experimentally predicted temperature change (6.02 K) from the FMR shift with a negligible error margin (see Supplementary Material S4). Furthermore, a 2.5 K temperature gradient was observed from CPW1 toward the cross-junction. A similar temperature increase and gradient were also observed from CPW3 toward the junction along the vertical bar, as shown in Fig. 3h. Because of the base temperature change, the wavelength of the SWs changed as the excitation frequency was fixed. Thus, the interference pattern of the two waves changed, and destructive interference occurred at a different phase from that at room temperature. Moreover, as the SWs generated at CPW1 and CPW3 propagated toward the cross-junction, they gradually moved from the low-temperature to the high-temperature region, and their wavelengths were gradually converted^28^. This temperature-gradient-induced wavelength shift may also contribute to the interference pattern shift. However, the temperature gradient until the cross-junction (where interference occurred) was significantly small compared with the base temperature increase. Moreover, the distance traveled by SWs is only a few times larger than their wavelength until the cross-junction. This is insufficient to cause a significant change in the wavelength in the accumulative process. We compared the wavelength, propagation distance, temperature difference, and wavelength change results with previously reported wavelength shifts^28^ and found only a 0.14% and 1.14% change in wavelength in the case of BVMSWs and MSSWs, respectively, which is insignificant. Therefore, we excluded the effects of temperature-gradient-induced wavelength change of the propagating SW wavelengths in further calculations. We later explained the interference pattern change with the reconfiguration of the thermal landscape in terms of the dispersion relation. Moreover, as seen in Fig. 2a, we observed a drastic change in the ON/OFF ratio (isolation ratio) between the constructive and destructive interference in the dB scale upon the application of current. The intensity difference between constructive and destructive interference attained a maximum of 21 dB at *J*_DC_ = 0 GA m^−2^, reducing gradually thereafter until it reached 4.5 dB at *J*_DC_ = 6.7 GA m^−2^, whereupon it began increasing gradually to reach 10 dB at *J*_DC_ = 10.5 GA m^−2^. This can be explained in terms of the difference of incoming SWs signal intensity from CPW1 and CPW3 as scattering parameters *S*_21_ and *S*_23_, respectively. Smaller difference between *S*_21_ and *S*_23_ intensity results in a higher isolation ratio, whereas higher difference between them results in a lower isolation ratio (see Supplementary Materials S6). The correlation between the isolation ratio and the *S*_21_ and *S*_23_ differences is elucidated further in Supplementary Material S6.

**Figure 4:**
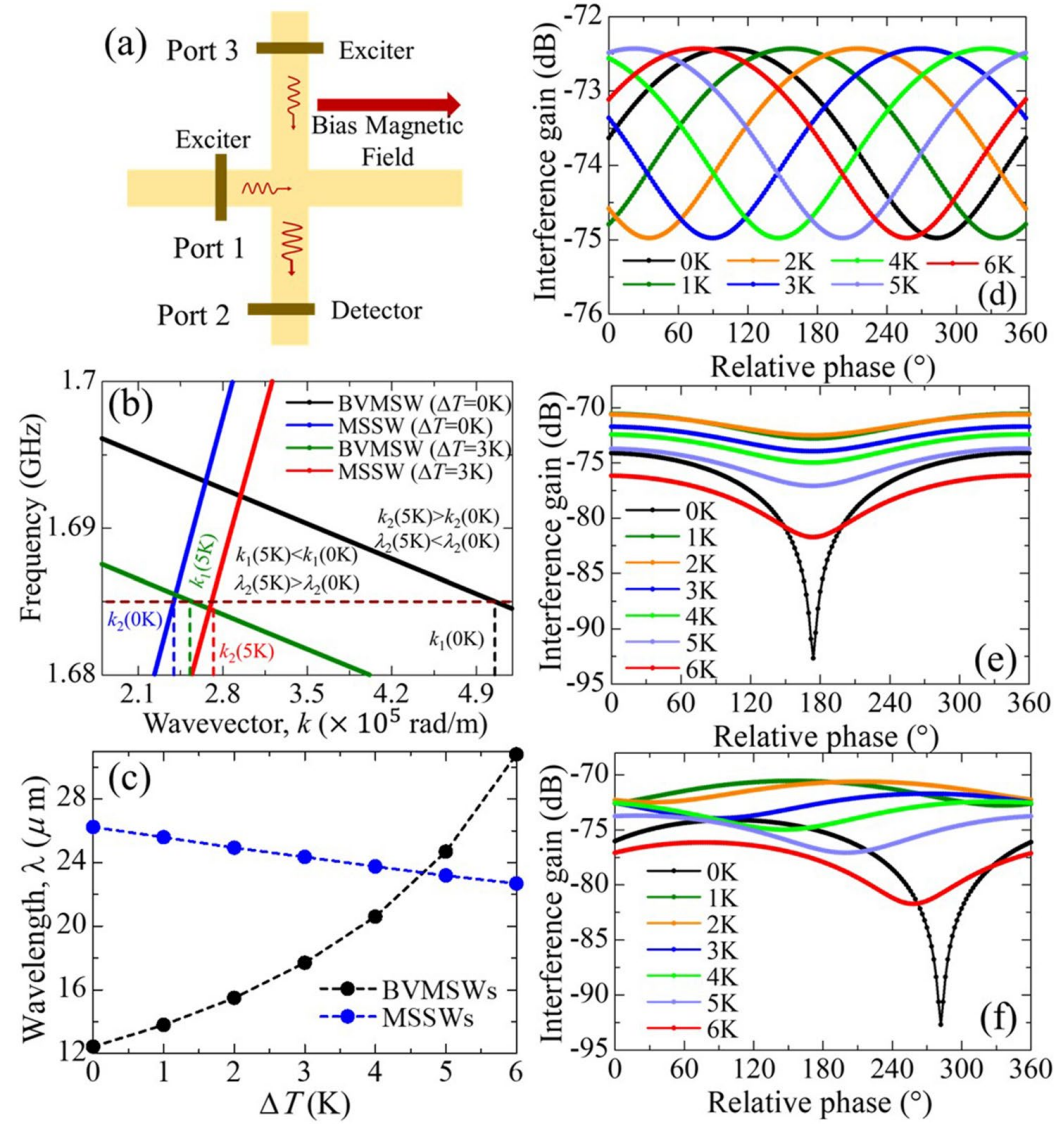
(**a**) Assumed structure for theoretical calculation. (**b**) Temperature-dependent dispersion relation. (**c**) Temperature-dependent wavelength change of MSSWs and BVMSWs. (**d**) Temperature-dependent interference pattern for fixed SW amplitudes. (**e**) Variable isolation ratio with varying SW amplitudes at different temperatures considering the constant wavevectors. SW amplitudes were extracted from the experimental data. (**f**) Interference pattern considering both phases and amplitude of the SW variables.

We built an interference model using MATLAB to explain the mechanism of the interference pattern change and to address the variation in the isolation ratio with temperature. For simulation, we reproduced the device on the same scale as that of the originally used in the above experiment, as shown in Fig. 4a. BVMSWs are excited by microwave pulse1 at Port 1, while MSSWs are excited by pulse 2 at Port 3. We used two plane-wave equations, ${M}_{\delta 1}={A}_{1}\mathrm{exp}\left[i\left({{\varvec{k}}}_{1}{\varvec{r}}+{\omega }_{1}t+{\varphi }_{1}\right)\right]$, and ${M}_{\delta 2}={A}_{2}\mathrm{exp}\left[i\left({{\varvec{k}}}_{2}{\varvec{r}}+{\omega }_{2}t+{\varphi }_{2}\right)\right]$, to depict the two waves excited at ports 1 and 3. The variable $\delta$ represents the magnetization component in a specific direction ($x, y,or z$), while $A,\boldsymbol{ }{\varvec{k}}, \omega, and \; \varphi$ represent the amplitude, wavevector, angular frequency, and phase, respectively. ${\varvec{r}}$ and $t$ are position and time, respectively. When ${\omega }_{1}={\omega }_{2}$, an effective and stable interference behavior is created. We set the wavevectors ${k}_{1}$ and ${k}_{2}$ based on the dispersion relation of BVMSWs and MSSWs, respectively. The value of ${A}_{1}$ and ${A}_{2}$ were set based on the experimental results (intensity of *S*_21_ and *S*_23_, respectively) presented in Supplementary Material S6. The phase difference $\Delta \mathrm{\varphi }=\left|{\varphi }_{1}-{\varphi }_{2}\right|$ was tuned from 0° to 360° at intervals of 2°.

To determine the specific values of wavevectors *k*_1_ and *k*_2_, we considered the effect of demagnetization field induced by the shape anisotropy, which have a significant influence on the dispersion relationships of BVMSWs and MSSWs. The dispersion relationship of BVMSWs is defined as^29^:

$$f(k)=\frac{1}{2\pi }\sqrt{{\omega }_{0}\left[{\omega }_{0}+{\omega }_{M}\left(\frac{1-{e}^{-kd}}{kd}\right)\right]}$$

However, the dispersion relationship of MSSW is described by^29^:

$$f(k)=\frac{1}{2\pi }\sqrt{{\omega }_{0}\left({\omega }_{0}+{\omega }_{M}\right)+{\left(\frac{{\omega }_{M}}{2}\right)}^{2}(1-{e}^{-2kd})}$$

where *d* is the thickness of the film, ${\omega }_{0}=\gamma {\varvec{H}}$, and ${\omega }_{M}=\gamma {M}_{s}$. Here ${\varvec{H}}$ is the effective field, including the external and anisotropy fields caused by the shape of the film and other factors, $\gamma$ is the gyromagnetic ratio, which is $2.21\times {10}^{5}$ m/(s A), and ${M}_{s}$ is the saturation magnetization. We set the thickness *d* as 90 nm and ${M}_{s}$ of YIG at room temperature as $1.4\times {10}^{5}$ A/m. The external field and shape anisotropy influence ${{\varvec{H}}}_{1}$ of the BVMSWs and ${{\varvec{H}}}_{2}$ of MSSWs were determined as follows. We first calculated the shape anisotropies by considering each YIG bar as a rectangular ferromagnetic prism, as explained in the literature^30,31^. The resulting calculated demagnetizing fields for the horizontal and vertical arms were $0.104\times {10}^{3}$ A/m and $0.753\times {10}^{3}$ A/m, respectively. Since the crossbar is a connected structure, it must have a single demagnetizing field^31^. A close approximation of the actual demagnetizing field can be obtained from the vector sum of the of the demagnetizing fields of the horizontal and vertical arms, which gives, ${H}_{shape} \cong 0.759\times {10}^{3}$ A/m. The demagnetization field increases the FMR frequency of BVMSWs and decreases the FMR frequency of MSSWs at the long wavelength limit^20^. Thus, we calculated ${H}_{1}={H}_{\mathrm{ext}}+{H}_{\mathrm{shape}}$ and ${H}_{2}={H}_{\mathrm{ext}}-{H}_{\mathrm{shape}}$, where the external bias magnetic field was ${H}_{ext}=18 \mathrm{mT}=1.43\times {10}^{4}$ A/m in the experiment.

The dispersion curves of the MSSW and BVMSW modes for a 5 K temperature change are represented by red and green curves (Fig. 4b), whereas they are represented by blue and black curves for the base temperature. The maroon dotted horizontal line indicates the fixed frequency of 1.685 GHz that was used in the interference measurement experiments. According to the dispersion relation, as the temperature increases 5 K above the room temperature, the wavevector of BVMSWs (*k*_1_) decreases from 0.506 to 0.255 rad/μm, resulting in a wavelength enhancement from 12.43 to 24.69 μm. Conversely, the wavevector of MSSWs (*k*_2_) increases from 0.240 to 0.271 rad/μm for the same temperature change, resulting in a wavelength reduction from 26.23 to 23.19 μm. Following the same technique, we calculated the dispersion curves of the two waves under different values of ${M}_{s}$ resulting from the film’s temperature change. We considered the temperature change from 0 to 6 K above the room temperature in 1 K intervals, its influence on the saturation magnetization *M*_s_(*T*), and the corresponding change in the wavevector and wavelength. The systematic change in the wavelengths of the BVMSWs and MSSWs upon thermal reconfiguration is shown in Fig. 4c.

We then sought to validate the contribution of thermal modulation to the phase shift of the destructive interference point. To do so, thermally modulated wavelength values were inserted to the plane-wave interference model and the phase position of the resulting destructive interference was recorded. We used the temperature-dependent wavevectors ${k}_{1}$ (BVMSWs) and ${k}_{2}$ (MSSWs), while the amplitudes of ${A}_{1}$ and ${A}_{2}$ were set to fixed values. The calculated interference pattern is shown in Fig. 4d. The shift in the interference pattern exhibited a similar trend to that observed in the experimental results shown in Fig. 2a. Here, the wavelength enhancement BVMSW is significant, and the wavelength becomes larger than that of MSSW at a 6 K temperature increase. This explains the almost complete cycle (360°) shift of the interference pattern resulting from a temperature change of 6 K in the experiment, compared to that at the base temperature. In the case of the 4 K (cyan) and 5 K (blue) curves, destructive interference occurred close to 180°, corresponding to perfect destructive interference. This is because the wavelengths of the interfering waves were identical at somewhere between 4 and 5 K, thus resulting in symmetric interference. In other cases, their wavelengths differ, resulting in an asymmetric interference where destructive interference does not occur at 180° phase difference. The calculation results of the temperature-induced interference pattern showed a uniform isolation ratio because we assumed fixed difference in values between A_1_ and A_2_. To address the amplitude-dependent change in the isolation ratio, as shown in Fig. 2a, we used the amplitudes of ${A}_{1}$ and ${A}_{2}$ extracted from the experimental data shown in Supplementary Material S4, where ${k}_{1}$ and ${k}_{2}$ are assumed to be the same. The calculation results of the temperature-induced interference pattern show different isolation ratios with identical destructive interference positions, as shown in Fig. 4e. In summary, these results demonstrates that the wavelength change of BVMSWs and MSSWs mainly contributes to the phase shift of the destructive interference point, while the isolation ratio is influenced by the amplitude difference of BVMSWs and MSSWs. We determined the interference pattern considering temperature dependent wavevector and SWs amplitude, as shown in Fig. 4f, which corresponds to the experiment results with a negligible error margin. The spin Seebeck effect (SSE) may induce SWs propagation along the temperature gradient of the YIG film. However, the propagation of SSE-induced SWs is incoherent. This study focuses on microwave-induced coherent SWs, ignoring the noncoherent part. Bracher et al.^32^, reported the generation of BVMSWs by the interference of two counter propagating MSSWs in a T-shaped magnonic microstructure. They suggested that the interference of two counter propagating MSSWs with identical wavevectors can generate a BVMSWs with a different wavevector. This also gives an intuition of the existence reverse effect, i.e., the interference of two SWs with non-identical wavevectors. The effect of dynamic modulation on this mode conversion process may be an interesting topic for future investigation.

## Reconfigurable logic

**Figure 5:**
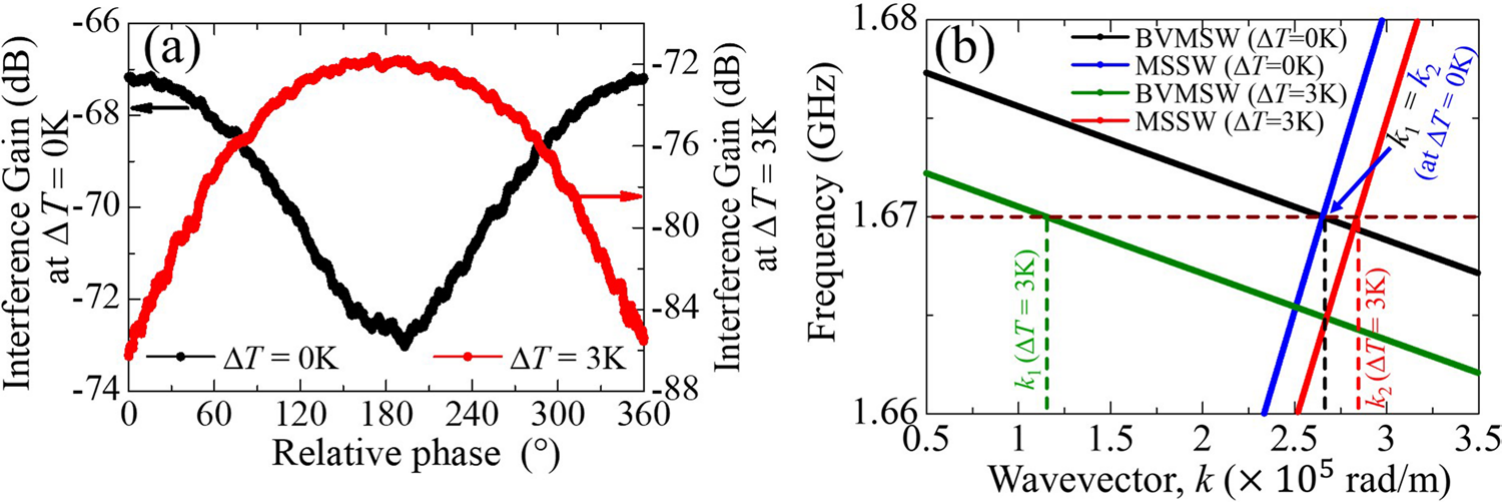
(**a**) Interference patterns at 0 K (black curve) and 3 K (red curve) above the room temperature. It demonstrates the reconfiguration from XNOR to XOR logic by altering the thermal landscape. (**b**) Corresponding dispersion curves of BVMSWs and MSSWs at different temperatures.

The external field-dependent wavelength conversion demonstrated above has some exciting potential applications. In this study, we focused on the potential of the tunable interference for application in a reconfigurable logic gate. It is proposed that such a logic gate could operate in XNOR or XOR modes, owing to the *J*_DC_-induced thermal restructuring enabling either symmetric or asymmetric interference. We previously concluded that the effect of the thermal gradient on the interference pattern is insignificant compared with the base temperature increase. Therefore, we increased the temperature of the entire device uniformly using a Peltier device, similar to that used by Alam et al.^33^ (see Supplementary Material S7). The magnetic field was the same as before; however, owing to the placement of the Peltier device below the substrate, the magnetization distribution changed, and the effective magnetic field was reduced to 17.6 mT at the sample location. The black and red curves in Fig. 5a correspond to the interference pattern at room temperature and at a temperature increase of 3 K above room temperature, respectively. The black and blue dispersion curves in Fig. 5b correspond to the room-temperature interference in Fig. 5a. Symmetric interference of the two identical wavevectors (0.266 rad/μm for both BVMSW and MSSW) occurred; therefore, we observed destructive interference at a phase difference of 180°. As the base temperature increased, the wavevectors of the BVMSWs and MSSWs changed from 0.266 rad/μm, decreasing to 0.115 rad/μm and increasing to 0.284 rad/μm, respectively. Therefore, the destructive interference pattern changed, shifting from 180° to 360° (supporting calculations are supplied in Supplementary Material S8). The black curve in Fig. 5a represents the XNOR gate, where the output is low when one of the inputs is out of phase with another. The red curve represents the XOR logic gates, where out-of-phase input results in high output logic. The detailed logic patterns are listed in Table 1.

**Table 1:** Reconfigurable XOR and XNOR logic table.

Port 1	Port 3	Port 2 (Δ*T* = 0 K)	Port 2 (Δ*T* = 3 K)
0 (0°)	0 (0°)	High	Low
0 (0°)	1 (180°)	Low	High
1 (180°)	0 (0°)	Low	High
1 (180°)	1 (180°)	High	Low

## Conclusion

In this study, we demonstrated asymmetric magnon interference using two different type of SW modes (BVMSWs and MSSWs), where each of the interfering SWs had different wavelengths. By controlling the difference in the wavelength, we showed that the destructive interference position could be controlled at any relative phase difference. We used on-chip current-induced changes in the thermal landscape to introduce such asymmetry. Two types of contributions, temperature rise and thermal gradient, were expected. However, considering the propagation distance and the amount of thermal gradient, the contribution of the thermal gradient to the SW wavelength conversion was shown to be negligible. By fine-tuning the symmetric and asymmetric interference, we propose that our mechanism can be used to implement a reconfigurable logic device that can operate between the XNOR (at Δ*T* = 0 K) and XOR (at Δ*T* = 3 K) logic modes via on-chip control technique using current-induced temperature changes. Our proposed technique can be easily translated to any controlling techniques (e.g., electric field- or strain-controlled techniques) to significantly reduce power consumption and increase reconfiguration speed. Careful design of operating point showed that only 3–5 MHz shift of the SWs frequency is necessary for the desired reconfigurability, which can be easily reached by voltage^34^ and strain^35^. Moreover, it was demonstrated that the isolation ratio of the proposed device could be changed using external control, owing to a change in the difference in the interfering signal intensity. By carefully designing the device dimensions, all interference cases can achieve a steady isolation ratio.

## Method

A 90 nm-thick YIG film was grown on a single-crystalline (001)-oriented gadolinium gallium garnet (Gd_3_Ga_5_O_12_, GGG) substrate using pulsed laser deposition. The temperature and oxygen pressure in the growth chamber were maintained at 750 °C and 0.1 Pa, respectively. An ArF excimer pulse was applied at a rate of 5 Hz. The films were annealed in air for 3 h at 800 °C. The crystallinity of the annealed thin film was confirmed via XRD by using a PANalytical Empyrean diffractometer in a $2\theta -\theta$ mode, as shown in Supplementary Material S1a. The clear Laue oscillation around the film peak indicates the high crystallinity of the film. Next, we conducted laser lithography (Heidelberg DWL66 +) and wet chemical etching with hot orthophosphoric acid at 140 °C to fabricate a cross structure. An optical image of the crossbar device is shown in Supplementary Material S1b. The length and width of the horizontal bar of the crossbar were 300 and 65 μm, respectively, whereas 450 and 40 μm for vertical bar. The inequality in the signal strength between BVMSWs and MSSWs SWs led to the asymmetric dimension. BVMSWs have a low intensity owing to its low excitation efficiency compared with MSSWs; thus, its propagation length is shorter than that of MSSWs. Therefore, we made the propagation distance of BVMSWs shorter than that of MSSWs to reach the cross-junction position to equalize the signal intensity for interference. Additionally, the accommodation of CPW antennas motivated us to design asymmetric crossbars.

Subsequently, alignment exposure was conducted to prepare a thin Pt layer on the right wing of the horizontal bar of the YIG crossbar. DC magneto-sputtering (ULVAC) was used to fabricate a 50 nm-thick Pt layer. This Pt layer functions as a local heater to increase the temperature of the crossbar locally. Another layer of lithography was used to pattern the CPWs to connect with the VNA ports and the DC electrodes to connect with the Pt heater. DC magneto-sputtering-grown 100 nm-thick Au was used to fabricate the CPWs and DC electrode structures. A 4 nm Cr layer was used as the adhesion layer between the Au and substrate. The edge-to-edge distance of the signal wires of the CPWs along the vertical bars (CPW2 and CPW3) was 175 μm. The distance from the signal wire of the GS waveguide to the nearest edge of the Pt heater along the horizontal bar was 60 μm. We used the G-S (Ground-Signal) coplanar waveguide in Port 1 (CPW1) instead of the G-S-G (Ground-Signal-Ground) coplanar waveguide in ports 2 and 3 (CPW2 and CPW3) to reduce the propagation distance of the BVMSW because of its low excitation efficiency. We also brought the signal wire of the antenna closer to the cross-junction. The length and width of the Pt heater were 122 μm and 65 μm, respectively. We prepared a Pt heating layer at the right-side edge of the horizontal bar to reduce the adverse SWs absorption effect by the Pt layer during propagation. Moreover, it was intended that the Pt layer may function an SWs damper at this location to reduce the signal reflection from the YIG bus edge.

## Supplementary Information

Supplementary Information.

## Data Availability

The data that support the findings of this study are available from the corresponding author upon reasonable request.
