# Supplementary material for: Reconfigurable magnon interference by on-chip dynamic wavelength conversion
Source: Sci Rep. 2023 Mar 24;13:4872. doi: 10.1038/s41598-023-31607-7 (PMC10039069; doi:10.1038/s41598-023-31607-7)
Supplement: Supplementary file 1 — Supplementary Information. [file 41598_2023_31607_MOESM1_ESM.pdf]

Supplementary Material

Reconfigurable magnon interference by on-chip dynamic wavelength conversion

Md Shamim Sarker[1,5*], Lihao Yao[1], Hiroyasu Yamahara[1,3*], Kaijie Ma[2], Zhiqiang Liao[1], Kenyu Terao[1], Siyi Tang[1], Sankar Ganesh Ramaraj[2], Munetoshi Seki[1,3,4], Hitoshi Tabata[1,2,3,4*]

[1] Department of Electrical Engineering and Information Systems, Graduate School of Engineering, The University of Tokyo, 7-3-1 Hongo, Bunkyo-ku, Tokyo 113-8656, Japan
[2] Department of Bioengineering, Graduate School of Engineering, The University of Tokyo, 7-3-1 Hongo, Bunkyo-ku, Tokyo 113-8656, Japan
[3]Institute of AI and beyond, The University of Tokyo, 7-3-1 Hongo, Bunkyo-ku, Tokyo 113-8656, Japan
[4]Center for Spintronics Research Network, Graduate School of Engineering, The University of Tokyo, 7-3-1 Hongo, Bunkyo-ku, Tokyo 113-8656, Japan
[5]Department of Electrical and Electronic Engineering, Khulna University of Engineering and Technology, Khulna-9203, Bangladesh
* tabata@bioeng.t.u-tokyo.ac.jp, yamahara@bioxide.t.u-tokyo.ac.jp, sarker@bioxide.t.u-tokyo.ac.jp

Supplementary Information:

S1. XRD scan of YIG film and optical image of the interference device

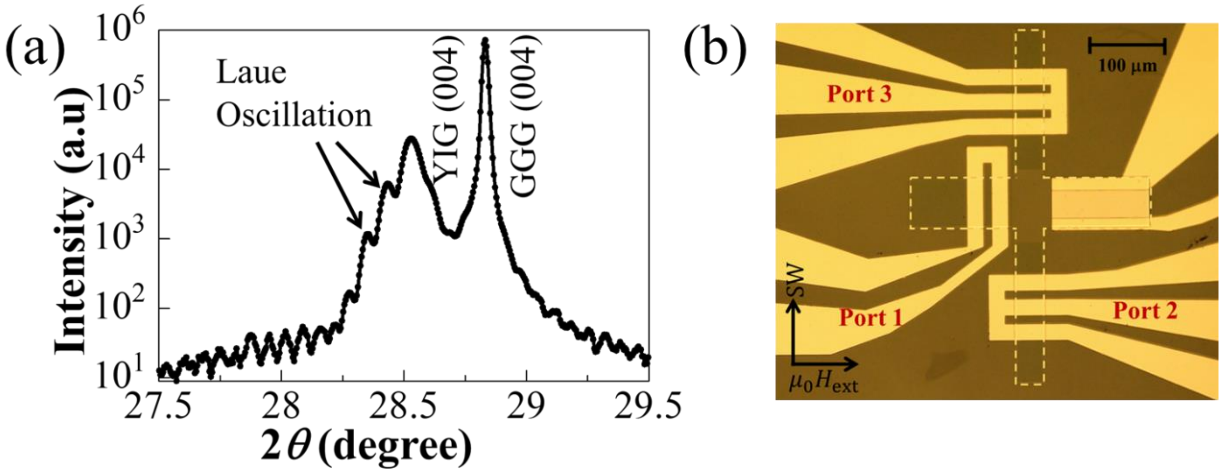

Figure S1. (a) $2\theta - \theta$ XRD scan of the PLD-grown YIG thin film (b) Optical image of the fabricated crossbar SWs interference device.

S2. J_{DC}-dependent interference pattern

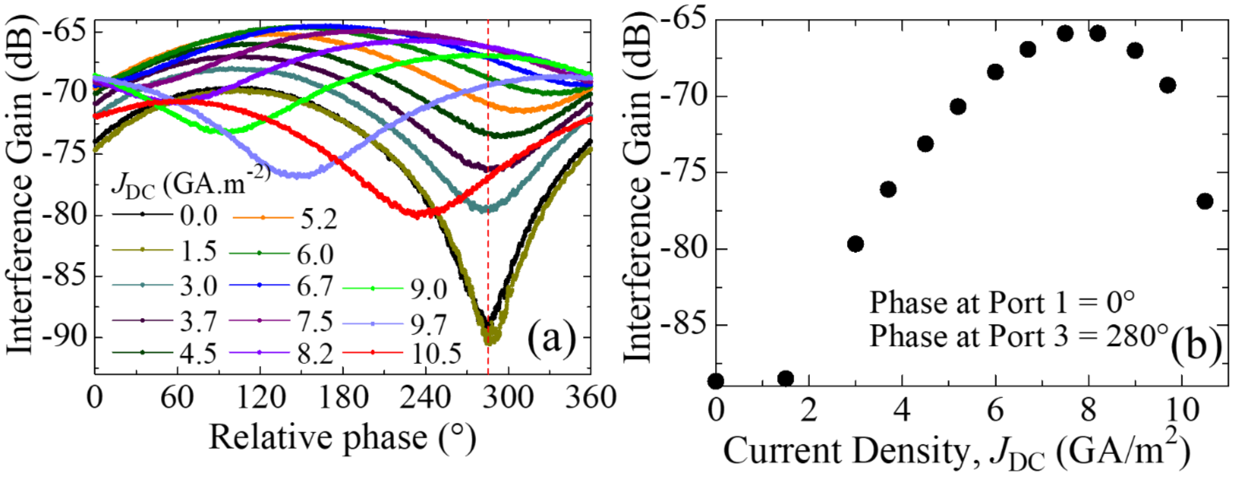

Figure S2. (a) Tunable interference pattern by the current-induced thermal landscape. (b) Distribution of J_{DC}-dependent interference gain at a fixed phase difference of 280°.

S3. Temperature-dependent magnetization of YIG thin film

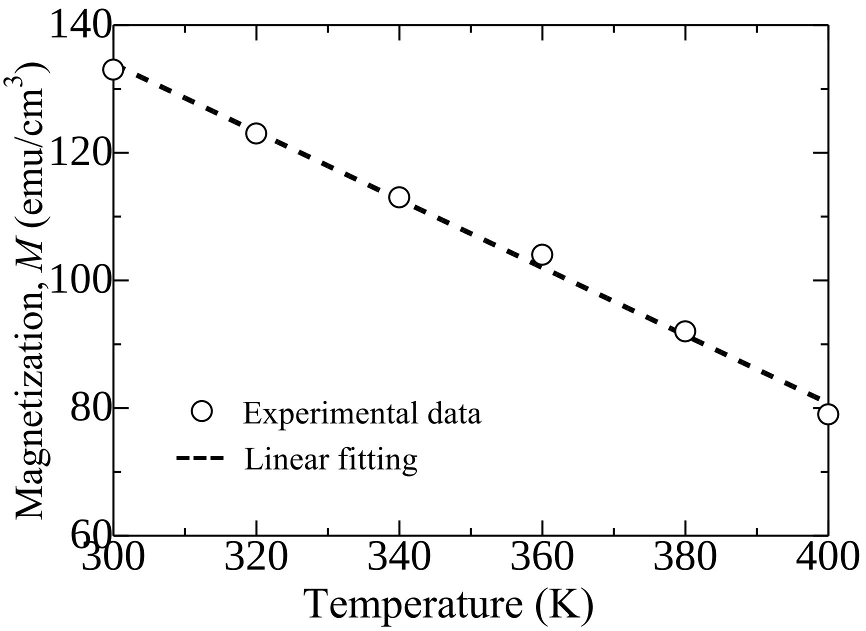

Figure S3. Temperature-dependent magnetization of YIG thin film exhibiting an inversely proportional, nearly-linear relationship between 300 to 400 K.

S4. Prediction of temperature change from the FMR shift

The current-dependent resonance frequency shift is shown in Figure S4(a). The open circles denote the experimental data, which were fitted (dotted line) with the temperature-dependent modified Kittel equation shown below.

$$f_{\mathrm{FMR}} = \frac{\gamma}{2\pi}\sqrt{H_{\mathrm{ext}}\left(H_{\mathrm{ext}} + M_{\mathrm{s,RT}} - \eta(T - T_{\mathrm{RT}})\right)}. \qquad (2)$$

Using this equation, we can express the temperature dependent f_{FMR} shift as $\Delta f_{\mathrm{FMR}}(T - T_{\mathrm{RT}}) \approx \Delta f_{\mathrm{FMR}}(\Delta T) = f_{\mathrm{FMR}}(T) - f_{\mathrm{FMR}}(T = T_{\mathrm{RT}})$. From this current dependent resonance shift, we calculated the temperature change as shown in Figure S4(b). To quantify the current-dependent temperature change, we measured the current-dependent resistance ($R_{(I)}$) of the Pt layer, shown in Figure S4(c) and found that it increases quadratically with applied current, according to $R_{(I)} = R_0 + R_2 I^2$, where $R_2 = 45\ \Omega A^{-2}$ is the fitting parameter and $R_0 = 9.3\ \Omega$ is the total resistance of the Pt layer at room temperature. Considering the thermal conductivity of YIG as 9 W/m/K and using the Wiedemann-Franz relation, Thiery *et. al.* analytically modeled the current-induced temperature change as $\Delta T = \kappa_{Pt}(R_{(I)} - R_0)/R_0$, where $\kappa_{Pt} = 254$ K is specific to Pt. Figure S4(b) represents the model-extracted ΔT as a function of I^2.The experiment and fitted curves agreed almost perfectly, and the temperature at the CPW1 region increased in steps of 0.1 K, 0.5 K, 1.1 K, 2.0 K, 3.0 K, 4.5 K, and 6.0 K upon the application of J_{DC} = 1.5, 3.0, 4.5, 6.0, 7.5, 9.0, and 10.5 GAm^{-2}, respectively.

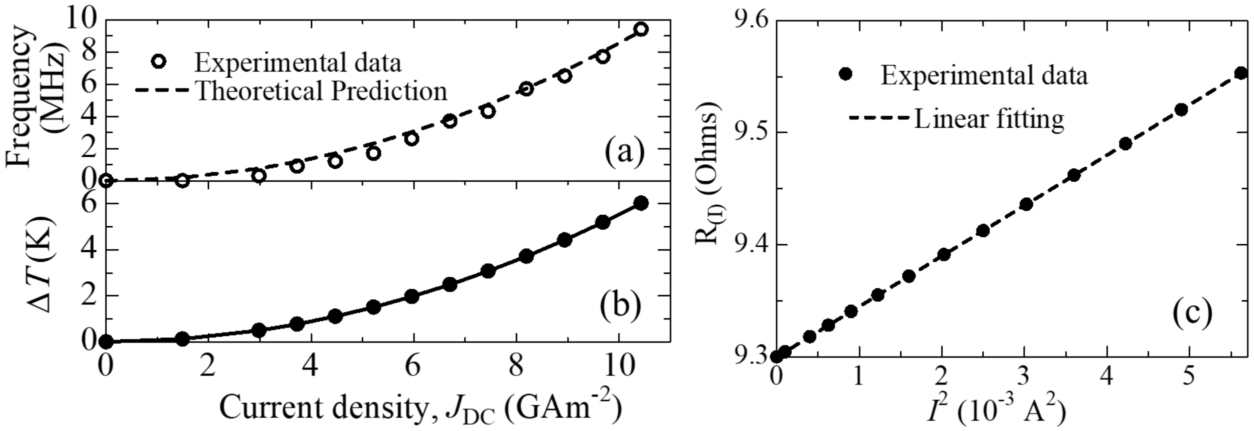

Figure S4: J_{DC}-dependent (a) frequency shift and (b) temperature change at CPW1. (c) Injected current (I^2)-dependent Pt stripe resistance.

S5. Generation of the thermal landscape by COMSOL

To map the temperature distribution of the crossbar SW device, the AC/DC module and heat transfer module in COMSOL Multiphysics were adopted for the simulation of heat generation and transfer. The simulated device comprised a crossbar YIG layer for SW propagation with a thickness of 90 nm on a GGG substrate (10 mm long, 10 mm wide, and 0.5 mm thick). Distances depicted in Figure S5 represent the seperations between antennas and heater. A Pt layer (50 nm thick) connected to an Au layer (90 nm thick) was deposited on the YIG, inducing Joule heating when an electric current flowed through it. When in operation, the upper side of the device was in contact with the surrounding air, and the bottom was in contact with the aluminum stage.

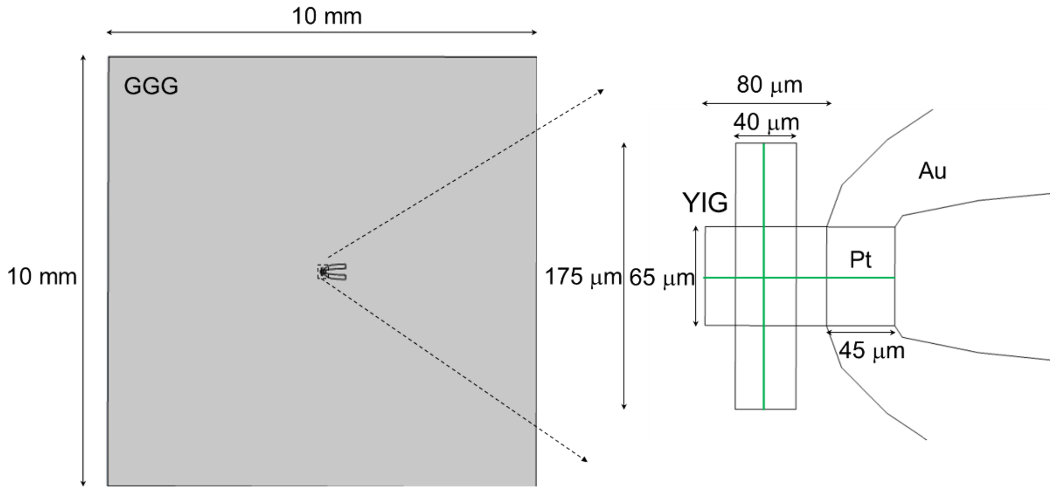

Figure S5. Physical model of heat generation and transfer module for COMSOL Multiphysics

The electric current and layered shell interface in the AC/DC Module were used to simulate the electrically-generated heat in the Pt layer when different electric currents (0 A, 0.005 A, 0.01 A, …, 0.065 A, 0.07 A) were applied. The produced heat rate per area in the Pt layer is defined as:

$$q = d \times Q = d \times (\vec{J} \cdot \vec{E}) = d\sigma(\nabla V)^2,$$

where d is the thickness of the Pt layer, Q is the power density, J is the current density, E is the electric field, σ is the electrical conductivity, and V is the voltage.

The heat transfer in the solid interface of the heat transfer module was used to simulate the heat transfer and temperature distribution in the entire device. After heat generation, the device dissipated heat to the surrounding air on its upper side and the aluminum stage on its bottom side, corresponding to heat flux in the convection process. The heat transfer in the device can be expressed as:

$$\rho C \vec{u} \cdot \nabla T + \vec{\nabla} \cdot (\overrightarrow{q_{conduction}} + \overrightarrow{q_{convection}}) = Q$$

$$-\vec{n} \cdot \overrightarrow{q_{convection}} = h(T_{ext} - T)$$

$$\overrightarrow{q_{conduction}} = -k\nabla T,$$

where ρ is the density, C is the heat capacity at constant pressure, $\vec{u}$ is the velocity vector of translational motion, T is the temperature, $\overrightarrow{q_{conduction}}$ is the heat flux by conduction, $\overrightarrow{q_{convection}}$ is the heat flux by convection, and Q is the power density of the heat source.

By coupling these two interfaces and setting the boundary conditions, the temperature distribution on the entire device can be obtained using the parameters listed in Supplementary Table 1.

Supplementary Table 1 Initial parameters in the simulation.

Parameter	Value	Parameter	Value
d_{Pt}	50 (nm)	σ_{Pt}	8.9×10^{6} (S m^{-1})
σ_{Au}	4.56×10^{7} (S m^{-1})	T_{air}	298.15 (K)
h_{air}	5 (W m^{-2} K^{-1})	T_{Al}	298.15 (K)
h_{Al}	239 (W m^{-2} K^{-1})	d_{YIG}	90 (nm)
d_{Au}	90 (nm)	ρ_{GGG}	7090 (kg m^{-3})
k_{GGG}	1 (W m^{-1} K^{-1})	C_{GGG}	371.75 (J kg^{-1} K^{-1})
C_{YIG}	590 (J kg^{-1} K^{-1})	ρ_{YIG}	5110 (kg m^{-3})
k_{YIG}	9 (W m^{-1} K^{-1})	C_{Pt}	133 (J kg^{-1} K^{-1})
ρ_{Pt}	21450 (kg m^{-3})	k_{Pt}	71.6 (W m^{-1} K^{-1})
C_{Au}	129 (J kg^{-1} K^{-1})	ρ_{Au}	19300 (kg m^{-3})
k_{Au}	317 (W m^{-1} K^{-1})		

S6. Explanation of isolation ratio change in terms of scattering parameters

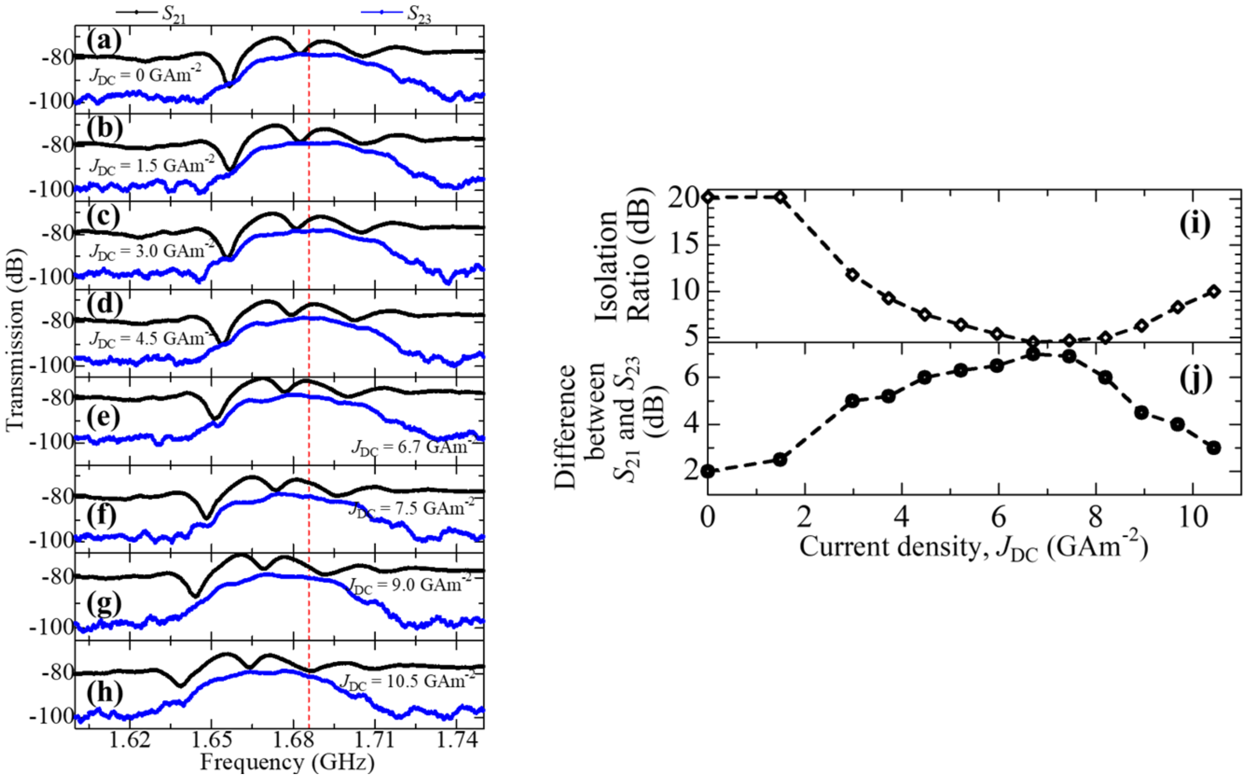

Figure S6. Scattering parameter, S_{21} and S_{23} at (a) J_{DC} = 0, (b) J_{DC} =1.5, (c) J_{DC} =3.0, (d) J_{DC} =4.5, (e) J_{DC} =6.7, (f) J_{DC} =7.5, (g) J_{DC} =9.0, and (h) J_{DC} =10.5 GAm^{-2}. J_{DC}-dependent (i) isolation ratio and (j) corresponding difference in signal intensity between two interacting signals.

From Figure 2(a) in the main text, we observed a drastic change in the ON/OFF ratio (isolation ratio) between the constructive and destructive interference in the dB scale upon application of current. The intensity difference attained a maximum of 21 dB at J_{DC} = 0 GAm^{-2}, reducing gradually thereafter until it reached 4.5 dB at J_{DC} = 6.7 GAm^{-2}. At this point, the difference increased gradually with J_{DC} and reached 10 dB at J_{DC} = 10.5 GAm^{-2}. This can be explained in terms of the difference in incoming SW signal intensity from CPW1 and CPW3, reflected in the scattering parameters S_{21} and S_{23}, respectively. As shown in Figure S4 (a–h), S_{21} and S_{23} represents the SW signals excited in CPW1 and CPW3, respectively, and detected in CPW2. According to the wave superposition theory, the intensity of the interfering waves should be equal to obtain the best contrast between constructive and destructive interference. We chose the 1.685 GHz frequency, as indicated by the red dotted line. As the current density increased, the intensity gap between S_{21} and S_{23} widened and reached a maximum difference of approximately 7.5 dB at J_{DC} = 6.7 GAm^{-2} owing to temperature-induced SW spectral shift. A larger gap between S_{21} and S_{23} indicates that a large signal is modulated by a small signal, which weakens the interference effect. Consequently, we observed a small difference in interference gain between constructive and destructive interference in the case of J_{DC} = 6.7 GAm^{-2}. However, with a further increase in the current density, the gap between S_{21} and S_{23} decreased to 3.5 dB, as shown in Figure 4(f–h).

Consequently, the ON/OFF ratio of the interference increased again, reaching 10 dB. The correlation between the differences between S_{21} and S_{23}, and the isolation ratio is illustrated in **Figure S6 (i–j)**.

S7. Proposed reconfigurable interference device under uniform heating by Peltier device

Our sample substrate was placed on a Peltier device to raise the temperature of the interference device uniformly, as shown in the figure below. In this way, the contribution of the temperature gradient was nullified.

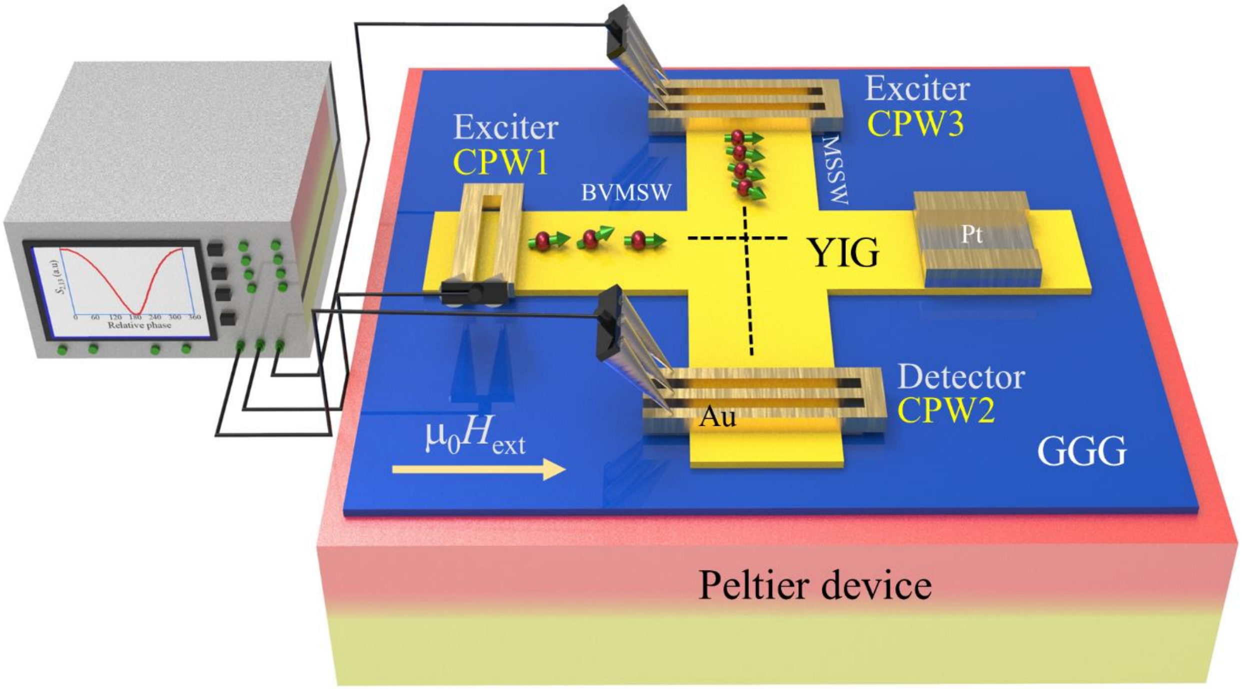

Figure S7: Reconfigurable interference device under uniform heating by Peltier device.

S8. Calculated reconfigurable interference mimicking XNOR and XOR

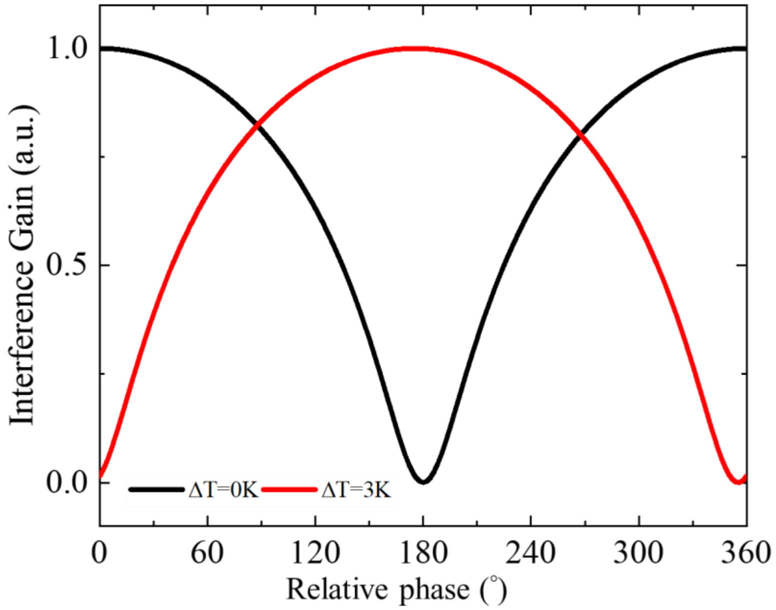

Figure S8. Calculated reconfigurable interference mimicking XNOR to XOR logic operation, considering a uniform temperature difference of 3 K.
